# Biomass carbon derived from pine nut shells decorated with NiO nanoflakes for enhanced microwave absorption properties†

**DOI:** 10.1039/c9ra00466a

**Published:** 2019-03-19

**Authors:** Huiya Wang, Yanlin Zhang, Qiuyue Wang, Chaowei Jia, Pan Cai, Gang Chen, Chengjun Dong, Hongtao Guan

**Affiliations:** School of Materials Science and Engineering, Yunnan University Kunming 650091 P. R. China dongchjun@hotmail.com htguan06@ynu.edu.cn +86-871-65037178; Yunnan Province Key Lab of Micro-Nano Materials and Technology, Yunnan University Kunming 650091 P. R. China

## Abstract

Electromagnetic absorption materials have gained increasing attention. In this study, we report NiO decorated biomass porous carbon derived from pine nut shells as a promising microwave absorbing material by a facile strategy. The NiO/biomass porous carbon (BPC) is thermally converted from Ni(OH)_2_/BPC with BPC as the base for precipitation. All products were characterized by XRD, Raman, and SEM techniques, which reveals that the NiO nanoflakes were uniformly self-assembled on the surface of the activated carbon. Compared with counterparts of pure Ni(OH)_2_ and Ni(OH)_2_/BPC, a large reflection loss peak of −33.8 dB at 16.4 GHz is achieved for the NiO/BPC composites, and the absorption bandwidth less than −10 dB can reach up to about 6.7 GHz (from 11.3 to 18.0 GHz) with a thickness of 8 mm. The enhanced microwave absorption properties originate from the electric/dielectric polarization and the unique NiO decorated BPC structures. The expanded interfaces, such as NiO–NiO, Ni–BPC and NiO–paraffin interfaces in the complicated porous composites, could boost the interfacial polarization as well as related relaxation which results in enhanced dielectric loss and electromagnetic absorbing properties. In addition, NiO/BPC nanocomposites exhibit comparatively better matching of permittivity and permeability. It is expected that our present investigation could provide a new possibility for biomass based fabrication of potential microwave absorbing materials.

## Abstract 2

A facile strategy was applied to synthesize NiO/biomass carbon composites with excellent microwave absorption properties.

## Graphical abstract

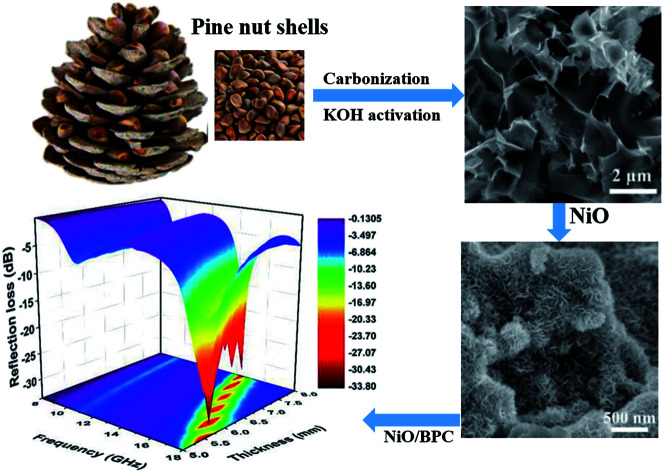

## 1. Introduction

Electromagnetic interference shielding and absorption are increasingly demanded due to the abundant electromagnetic applications in medical devices, sensitive equipment and mobile communications in both civil and military fields.^1^ The shielding or absorption performances are largely dependent on the interaction between the electromagnetic radiation and the materials, which is determined by the interior electromagnetic mechanism of the materials, such as interfacial polarization and spin relaxation, as well as resonance absorption.^2^ According to classic electromagnetic theories, electromagnetic absorbing materials (EAMs) can be sorted into three categories, *i.e.* electrical loss, dielectric loss and magnetic loss materials.^3^ To achieve better absorption, multiple loss mechanisms need to be built in the EAMs to fulfil the demands of high absorption and wide effective absorption bandwidth (EAB, the bandwidth with an absorption superior to −10 dB). In this sense, the physical characteristics, crystalline structures and morphological microstructures of the EAMs have great effects on their absorbing performances.

In the past few decades, researchers have focused great attention on improving the absorption properties and broadening their absorption bandwidth. Carbon based materials (carbon nanotubes (CNTs), carbon nanocoils and graphene),^4–8^ and transition metal related materials such as metal particles (Fe, Co, Ni and their alloys),^9–13^ oxides and hydroxides (CoO, Co_3_O_4_, NiO, MnO_2_, Fe_2_O_3_, Fe_3_O_4_, Ni(OH)_2_, MnOOH, *et al.*),^14–19^ sulfides (CuS, CoS) and phosphides (FeP, Co_2_P)^20–22^ have demonstrated excellent microwave absorption properties at certain frequency band with an optimum thickness. However, due to its electromagnetic loss mechanism, a single absorbent can hardly achieve a better performance in a wider frequency range and usually can only be used in some certain frequency. To get a comprehensive absorption performance with better absorbing values and wider frequency ranges, a best way is to be combined with other fillings to form corporate absorbents.

As a sort of transition metal materials, nickel oxides (NiO) and hydroxides (Ni(OH)_2_) have been extensively studied in the fields of magnetic and electrochemical applications. For example, Cooper *et al.* found that there were two magnetic transitions in NiO nanoparticles in the temperature range of 10 K to 400 K. At around 350 K, the NiO nanoparticles transform from paramagnetic form to superparamagnetism due to the surface defect effects.^23^ Similar phenomena have also been observed in Ni(OH)_2_ nanostructures.^24,25^ Sugiyama *et al.* discovered ferromagnetic dislocations in NiO films, which originate from the local non-stoichiometry of the dislocation cores with Ni deficiency.^26^ The dislocations could be a source of unique magnetic properties and may endow NiO with unexpected electromagnetic properties.^27^ To date, NiO and Ni(OH)_2_ are seldom applied solely for microwave absorption because of their weak magnetic loss and electrical conductivity. Zhang and co-authors synthesized reduced graphene oxide/NiO composites (RGO/NiO) by a pyrolyzation process and discussed their microwave absorption properties in the frequency range of 2–18 GHz.^28^ The NiO nanoparticles with sizes 10–50 nm are dispersing in the RGO sheets, resulting in a large microwave absorption peak of −55 dB and a wider EAB of 6.7 GHz. The excellent absorption performances can be ascribed to the increased dielectric loss, as well as the dipoles and defects after the introducing of RGO.^28^ Butera and Sakamoto gave new insights into the Fe doped Ni(OH)_2_ through density functional theory (DFT) calculations. They found that the electron occupied states of Ni(OH)_2_ after Fe doping presented great changes because of the delocalized nature of the band edges states, and thus would provide great influences on its electromagnetic and electrocatalytic performances.^25,29^

Recently, carbonaceous microwave absorbers have attracted intensive attentions due to their low density and better electrical conductivity. Among them, biomass derived porous carbon (BPC) materials, making from biomass wastes through carbonization and activation, have caught especial interests with the advantage of numerous natural sources, high porosity and synthesis facility.^30–38^ Till now, porous carbon synthesized from biomass sources such as willow catkins,^30,31^ plant leaves^32–34^ and kitchen foods or wastes^35–38^ with a subsequent activation show great conductivity and excellent electrochemical performances. In electromagnetic applications, BPC structures or their composites also display great electromagnetic interference (EMI) shielding or absorption properties.^38–41^ For instance, Naeem *et al.* developed porous carbon web from acrylic fibrous wastes, which revealed an EMI shielding effectiveness (SE) as high as 60–70 dB in the frequency range below 1.5 GHz.^39^ The high SE can be attributed to the increased multiple internal reflections and stronger absorption mechanisms. Yuan's group prepared lightweight and stiff carbon foam from flour and bread with carbonization and activation process and show a SE of about 17 dB in the frequency of 6–12.4 GHz.^40^ Chung *et al.* discussed the EMI shielding performance of carbon nanofiber (CNF) mats obtained with paper-making process, and found a SE value as high as 50–80 dB at 1.5 GHz with a thickness of about 5 mm.^41^ Their results show that the great SE can be attributed to the higher degree of three-dimensional electrical connectivity which enables the incident wave to reflect at a greater depth into the mat. Gong *et al.* prepared BPC with waste cotton and found a high electromagnetic absorption with a peak value of −40.5 dB at 15.8 GHz and an EAB of 6.76 GHz with 15% Ni filling, while the thickness is only about 1.9 mm.^42^

Pure carbon materials can deliver both shielding and absorption properties, which merely arises from electric loss and are determined crucially by their crystalline and morphological microstructures. To achieve an excellent absorbing performance, carbon materials often need series of fine-tuning processes to meet the basic requirements of impendence matching and good attenuation. To alleviate this dilemma, incorporation of carbon materials of electric loss along with magnetic or dielectric fillers has been considered as an effective strategy.^43^ Inspired by these pioneer works, combining nickel oxides or hydroxides with proper BPC materials will possess better dielectric and electrical properties and thus may be considered for electromagnetic absorbing application. In this work, using pine nut shells as the precursor, we developed a facile approach to design Ni(OH)_2_/BPC and NiO/BPC composites for EAM applications, which exhibits excellent absorption performances in the frequency range of X and Ku band (8–18 GHz). Based on the thorough characterizations, the possible absorption mechanisms are discussed as well.

## 2. Experimental

### 2.1 Materials and reagents

Pine nut shells were ground to powders, which were further passed through 140 mesh. Potassium hydroxide (KOH), hydrochloric acid (HCl), nickel nitrate (Ni(NO_3_)_2_·6H_2_O) and urea (CH_4_N_2_O) were purchased from Aladdin Chemicals Co. Ltd (Shanghai). Deionized (DI) water was used throughout the work.

### 2.2 Fabrication of biomass carbon and NiO/BPC composites

The biomass carbon was obtained by thermally treating the pin nut shells powders with KOH. Typically, the pin nut shells powders were sequentially rinsed with acetone, DI water and absolute alcohol, respectively. After dried at 80 °C for 10 h, the biomass was ground uniformly with KOH in a weight ratio of 1 : 2 in an agate mortar. Next, the composite was transferred into a furnace and calcined at 800 °C for 2 h under a N_2_ atmosphere, with a heating rate of 5 °C min^−1^. After cool down to room temperature, the BPC was thoroughly washed with 1 M HCl solution and DI water until its pH value reached about 7. The BPC was then dried at 80 °C for 10 h and collected for following use.

NiO/BPC composites were converted from Ni(OH)_2_/BPC. To synthesize Ni(OH)_2_/BPC, we ultrasonically dispersed 0.1 g BPC into 100 ml DI water. After that, 3.6 g Ni(NO_3_)_2_·6H_2_O was added into the solution and magnetically stirred for 4 h. Subsequently, 20 g urea was further introduced into the mixture and then kept in a water bath for 2 h with the temperature at 80 °C. When the reaction was completed, the precipitation was washed and centrifuged with DI water and ethanol for several times. Finally, Ni(OH)_2_/BPC composites were thus obtained. The Ni(OH)_2_/BPC was then calcined at 300 °C for 2 h under a N_2_ atmosphere to produce NiO/BPC. For comparison, intrinsic Ni(OH)_2_ and NiO were also prepared with the same procedure except for the introduction of carbon.

### 2.3 Characterization

Crystalline structures of the products were checked using X-ray powder diffraction (XRD) on a Rigaku TTR-III diffractometer with the Cu Kα radiation (*λ* = 0.15418 nm) in the diffraction range of 2*θ* = 10–80°. Raman spectra were recorded on a Renishaw inVia Raman microscope at room temperature with a 514 nm wavelength laser excitation and a power output of 3 mW. The morphologies of the samples were observed by scanning electron microscopy (SEM, FEI Nova NanoSEM 450 and Hitachi S-4800). The corresponding elemental mapping images were investigated by a FEI QUANTA 200 equipped with an EDX attachment at an accelerating voltage of 30 kV. Nitrogen adsorption–desorption isotherms were recorded in a specific surface area analyser (Quadrasorb-evo TM). The specific surface area was calculated from N_2_ adsorption isotherms using the Brunauer–Emmett–Teller (BET) equation, and the pore size distribution was estimated according to the Horvath–Kawazoe (HK) theory. The total pore volume was determined at a relative pressure of *P*/*P*_0_ = 0.98875. The thermal analysis in air was determined by means of an American TA SDT-2960 thermal analyzer with a heating rate of 5 °C min^−1^. The magnetic measurements were carried out on vibrating sample magnetometer (Lake Shore 7410).

The dielectric permittivity and magnetic permeability, as well as the electromagnetic *S* parameters (*S*_11_, *S*_12_, *S*_21_ and *S*_22_) were measured at room temperature from 8 to 18 GHz by a vector network analyzer (VNA, Agilent Technologies, N5230A). Before test, the mixtures were prepared by homogeneously adding the as-prepared products into paraffin wax in mass ratios of 30 wt%. Then the mixtures were shaped into a toroid with 2.00 mm in thickness, 3.04 mm in inner diameter, and 7.00 mm in outer diameter.

## 3. Results and discussion

### 3.1 Phase crystallinity

**Fig. 1:**
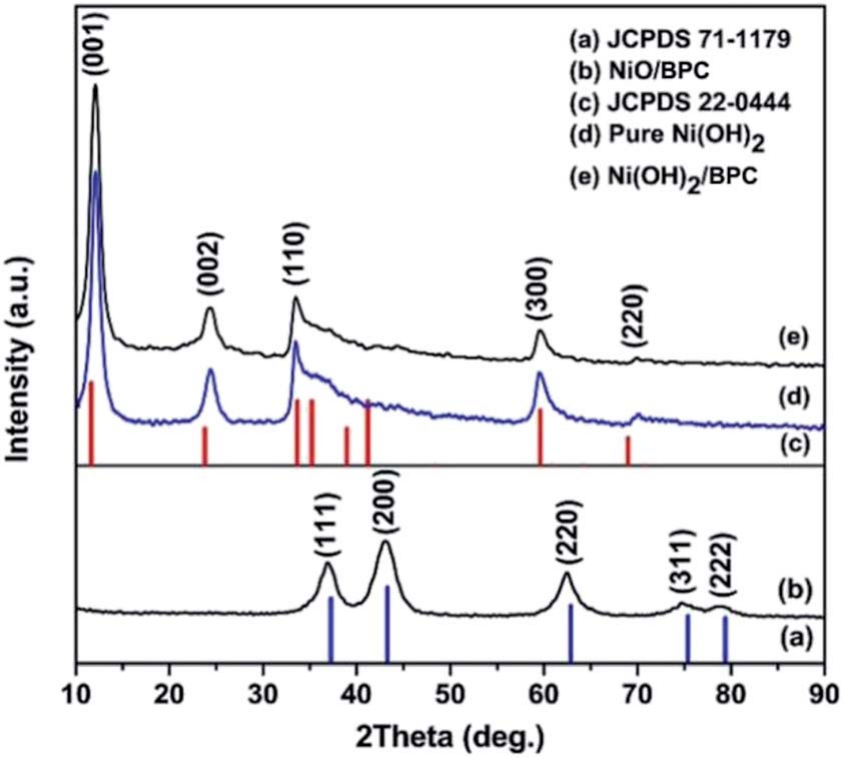
XRD patterns of the pure Ni(OH)_2_ (d), Ni(OH)_2_/BPC (e) and NiO/BPC (b) composites. (a) and (c) are the standard diffraction patterns of NiO and Ni(OH)_2_, respectively.

The phase and purity of the products were characterized by XRD and the patterns are presented in Fig. 1. For Ni(OH)_2_ and Ni(OH)_2_/BPC, all diffraction peaks (Fig. 1(d) and (e)) can be indexed to Ni(OH)_2_ (JCPDS 22-0444) (Fig. 1(c)) with a space group *P*3̄1*m* (162), suggesting a successful formation of Ni(OH)_2_. Regarding to the NiO/BPC composites (Fig. 1(b)), five major diffraction peaks related to the (111), (200), (220), (311), (222) crystal facet are observed, which is in good agreement with the cubic NiO pattern (JCPDS 71-1109) (Fig. 1(a)). Besides, no impurity peaks are found, evidently proving that the products are highly purified. It is worthy to note that the diffraction peaks of carbon are not visible due to the low contents and amorphous characteristics.

**Fig. 2:**
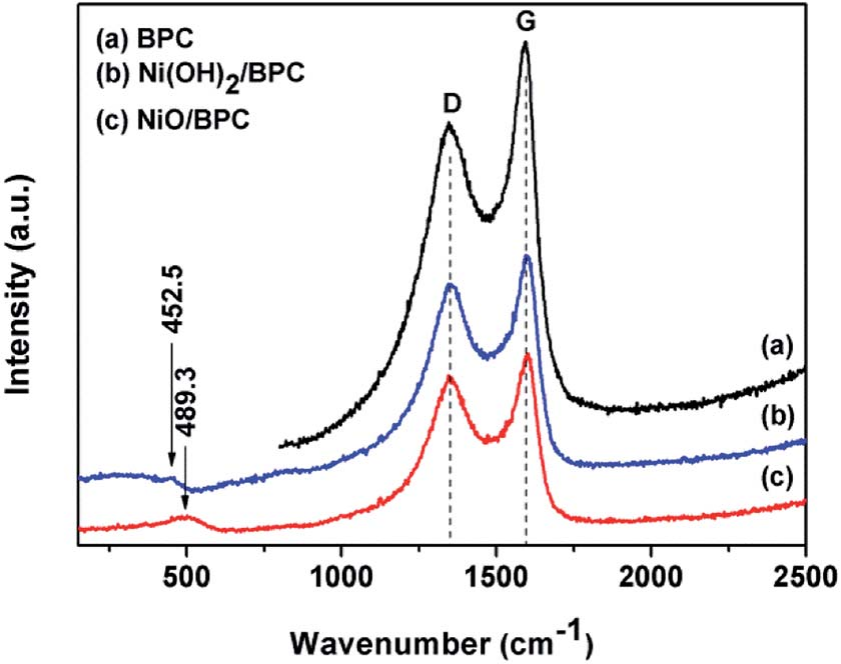
Raman spectra of the BPC (a), Ni(OH)_2_/BPC (b) and NiO/BPC (c) composites.

The Raman spectroscopy was also used to further investigate the compositions. As shown in Fig. 2, Raman spectra exhibit two peaks at 1345.9 cm^−1^ and 1594.8 cm^−1^, corresponding to the D band for disordered graphite carbon and G band representing the radial C–C stretching vibration mode of sp^2^ bond carbon, respectively.^44,45^ Compared with pure BPC, the integrated intensity ratio of D and G bands (*I*_D_/*I*_G_) changes from 0.85 for Ni(OH)_2_/BPC to 0.91 of NiO/BPC, indicating a higher degree of the defects in the graphitized structure or the edges. These defects could act as polarized centres for the dipole/electrons polarization, which may be contributed to enhance the dielectric loss and electromagnetic energy dissipation by reason of Debye relaxation. Debye relaxation occurs when the dipole and electrons polarizations could not match up with the changes of the electromagnetic field in the high frequency,^46,47^ which plays an import role in microwave absorbing and shielding. Furthermore, two broad humps at 452.5 cm^−1^ (Fig. 2(b)) and 489.3 cm^−1^ (Fig. 2(c)) can be assigned to the Ni–OH stretching mode (Ni(OH)_2_/BPC) and the vibrations of the Ni–O stretching (NiO/BPC), respectively.^48,49^

The above results strongly indicate that the Ni(OH)_2_ is efficiently assembled on the surface of BPC, thus the NiO/BPC composites were formed after thermal treatment. The conversion from Ni(OH)_2_/BPC to NiO/BPC was examined by the simultaneous TGA and DSC analysis and the results were illustrated in Fig. S1.† It is found that the total weight loss of Ni(OH)_2_ was measured to be 34.55%, as in Fig. S1(a).† Apart from the removal of the physically adsorbed water molecules, a big weight loss is observed around 300 °C. Meanwhile, the DSC curve shows an endothermic peak with a maximum located at 298.23 °C, corresponding to endothermic behaviour during the decomposition of Ni(OH)_2_ to NiO. In the case of Ni(OH)_2_/BPC (Fig. S1(b)†), the first descent stage below 241 °C is caused by the adsorbed water which loses about 10.20% in total weight. The second weight loss of 17.05% is attributed to the transformation of Ni(OH)_2_ to NiO. Next, the weight variation above 340 °C is derived from the change of biomass activated carbon to CO_2_ in the air, which accounts for 21.11% of the total weight. Accordingly, two endothermic peaks are observed at 313.37 °C and 453.21 °C. Thus, the content of Ni(OH)_2_ is estimated to be 71.60% in the Ni(OH)_2_/BPC composites. Similarly, the mass fraction of NiO can be determined to be around 67.80% in the NiO/BPC, as shown in Fig. S1(c).†

### 3.2 Morphological structures

The morphological structures of all products including Ni(OH)_2_, BPC, Ni(OH)_2_/BPC, and NiO/BPC are analysed by SEM measurements. For the as-prepared pure Ni(OH)_2_, the Ni(OH)_2_ nanosheets are proved to be interconnected together to form spherical structures with a diameter of about 2 μm (Fig. S2(a)†). Magnified image (Fig. S2(b)†) reveals that the Ni(OH)_2_ exhibits a honeycomb-like structure with randomly dispersed silk build blocks on the surface. The introduction of activated carbon will greatly impact the growth of Ni(OH)_2_.

**Fig. 3:**
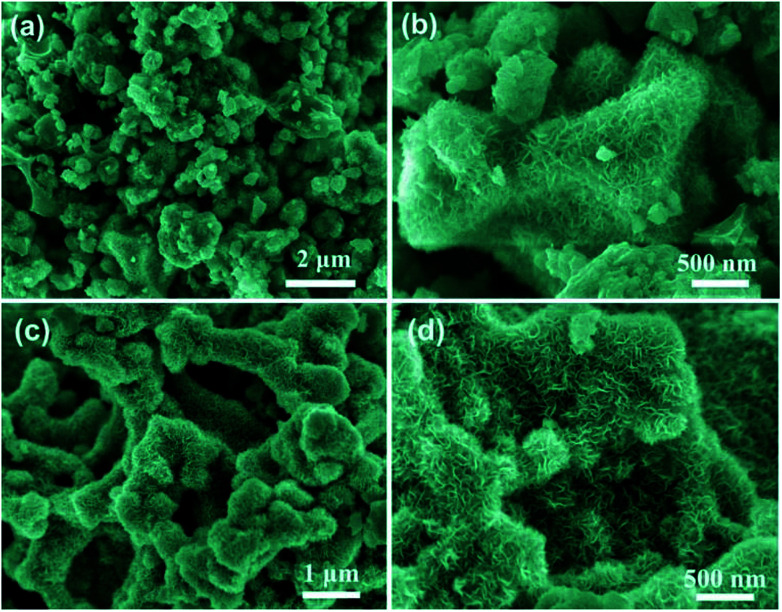
SEM images of Ni(OH)_2_/BPC (a and b) and NiO/BPC composites (c and d).

KOH activation plays a great role in the formation of the unique porous by etching the carbon matrix through a redox reaction to produce abundant micro/mesoporous structures.^36^ The intermediate products (metal K) can intercalate in the carbon framework and expand the carbon lattices, and thus leading to the porous structure.^50^ After activated by KOH, the pine nut shells derived biomass carbon has been broken into small particle clusters, which presents both loose and porous structures with particles from 7–15 μm in size (Fig. S2(c)†). It can be clearly seen from Fig. S2(d)† that the activated carbon appears a large number of interconnected holes with uneven size. This porous structure will favour the improvement of specific surface areas, thus providing sufficient sites for Ni(OH)_2_ nanosheets distribution. Combining BPC with Ni(OH)_2_, the activated carbon are densely packed by a large number of extremely thin and rippled nanosheets with similar arc-shaped morphologies, thus forming the multi-layered porous structure (Fig. 3(a and b)). Such structure can alleviate nanoflakes stacking, promote the formation of interparticle spacing, and increase specific surface area.^51,52^ After thermally converted to NiO/BPC, the basic microstructures of Ni(OH)_2_/BPC are preserved well. Moreover, well defined contour seems to be observed for NiO/BPC due to the recrystallization of NiO.

**Fig. 4:**
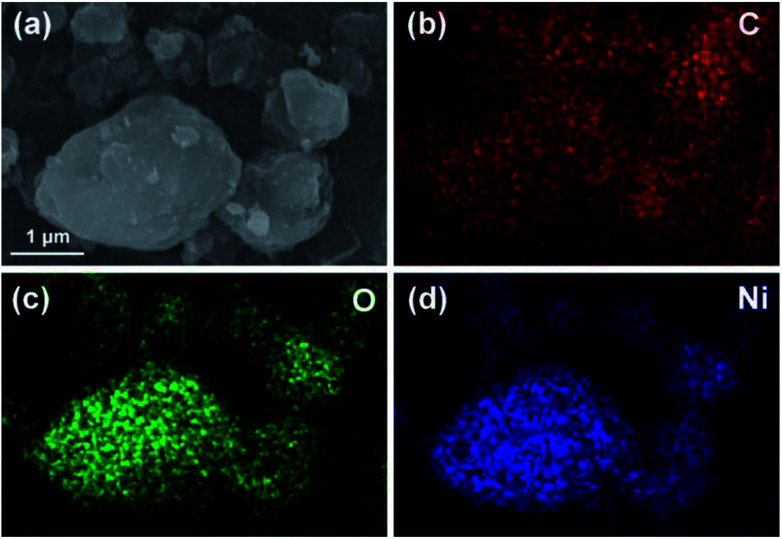
SEM image of the NiO/BPC composite for elemental mapping (a), and the corresponding elemental distributions of C (b), O (c) and Ni (d).

To further illuminate the distribution of the elements, an element mapping was performed by taking NiO/BPC composite as an example. As shown in Fig. 4, the elements of C, O and Ni demonstrate a very homogeneous distribution, which further verifies the NiO nanosheets are uniformly decorated on the surface of activated carbon. According to traditional electromagnetic theories, the nanosheet-like structures along with the carbon compositions, make the Ni(OH)_2_ and NiO easily to be polarized, thus will endow them potential electromagnetic functionalities.

**Fig. 5:**
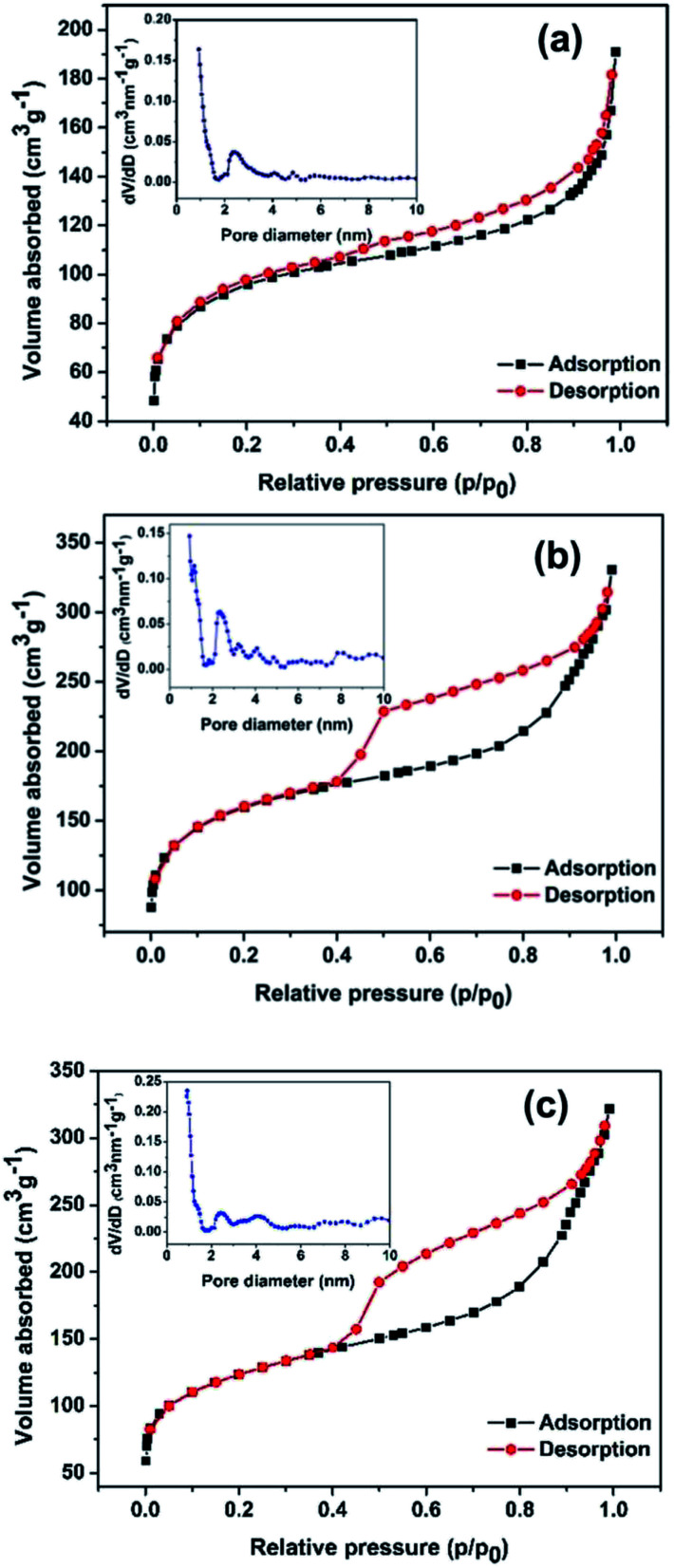
Nitrogen adsorption–desorption isotherms and the pore diameter distribution inserted of BPC (a), Ni(OH)_2_/BPC (b) and NiO/BPC (c) composites.

The nitrogen adsorption–desorption isotherms are generally employed to characterize the specific surface area and pore structures of porous materials. In the present work, the as-synthesized BPC, Ni(OH)_2_/BPC and NiO/BPC composites were analysed through nitrogen adsorption–desorption and the results are displayed in Fig. 5. As demonstrated in Fig. 5, both the BPC and the composites present a representative type-II isotherm with a H_3_ hysteresis loop, suggesting a micro/mesoporous structure. The sharp rise of nitrogen uptake capacity at the low pressure from 0 to 0.1 mainly results from micropore adsorption. The subsequent increase in nitrogen uptake capacity illustrates the existence of mesoporous structures. Specifically, the specific surface area of 346.15 m^2^ g^−1^ and the total pore volumes of 0.296 m^3^ g^−1^ are obtained for activated biomass carbon, as shown in Fig. 5(a). However, a higher specific surface area of 574.35 m^2^ g^−1^ and 440.45 m^2^ g^−1^ are calculated for Ni(OH)_2_/BPC (Fig. 5(b)) and NiO/BPC (Fig. 5(c)) composites, respectively, with similar average pore distribution centered at 0.93 nm and 0.89 nm, as shown in Fig. 5(b) and (c). These special cavities with high surface areas and small pore sizes may effectively trap microwaves and form abundant interfaces and junction areas, which could lead to the aggregating charges bound and cause the interfacial polarization as well as related relaxation.^53^

### 3.3 Magnetic properties

**Fig. 6:**
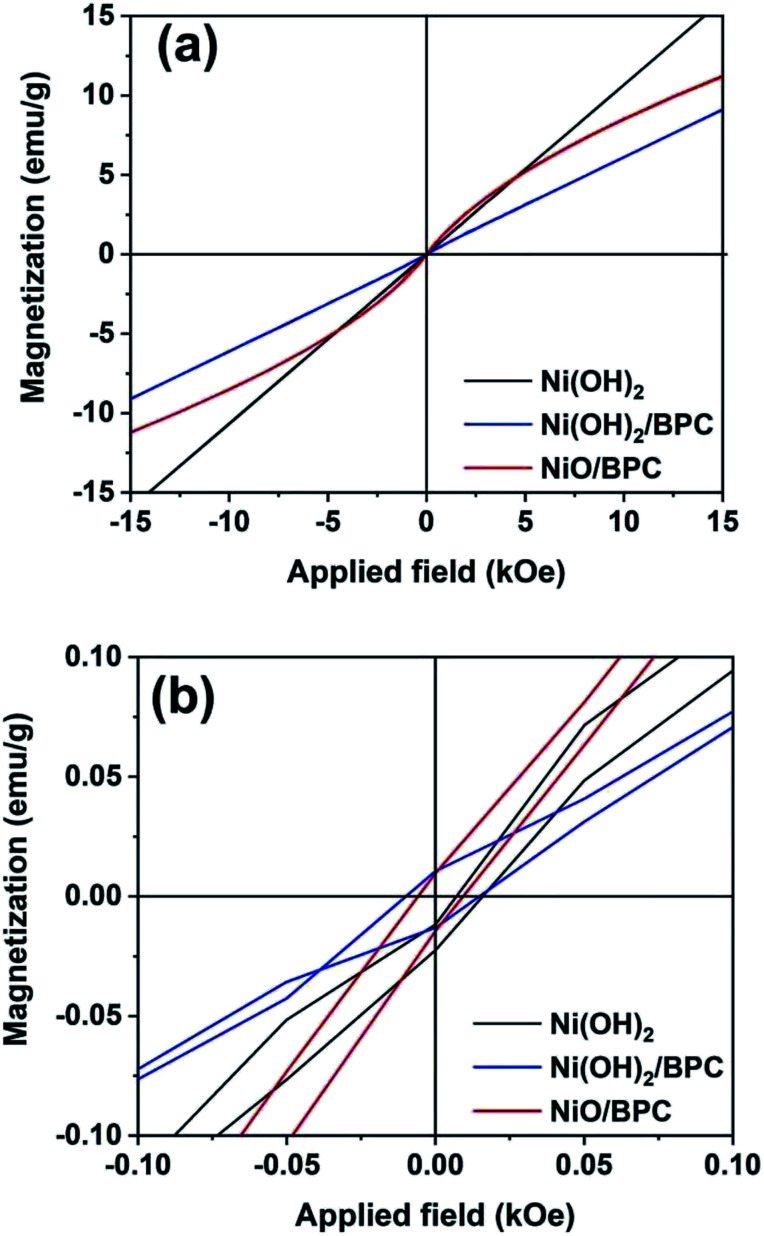
Magnetic hysteresis loops of Ni(OH)_2_, Ni(OH)_2_/BPC and NiO/BPC composites (a) and their magnification view for coercivity (b).

Magnetic hysteresis loops of the three samples were measured by vibrating sample magnetometer (VSM) at room temperature to investigate the magnetic properties, as shown in Fig. 6. Both pure Ni(OH)_2_ and Ni(OH)_2_/BPC demonstrate the hysteresis loops as almost straight lines with their coercivity of almost zero, indicating much lower ferromagnetic characteristics. The results are in line with those reported by Tiwari.^54^ For NiO/BPC composite, it shows a semi-S shape hysteresis loop, suggesting a ferromagnetic attribution. However, the nonsaturated hysteresis loop and almost zero coercivity also indicate its weak magnetism.

### 3.4 Microwave absorption properties

**Fig. 7:**
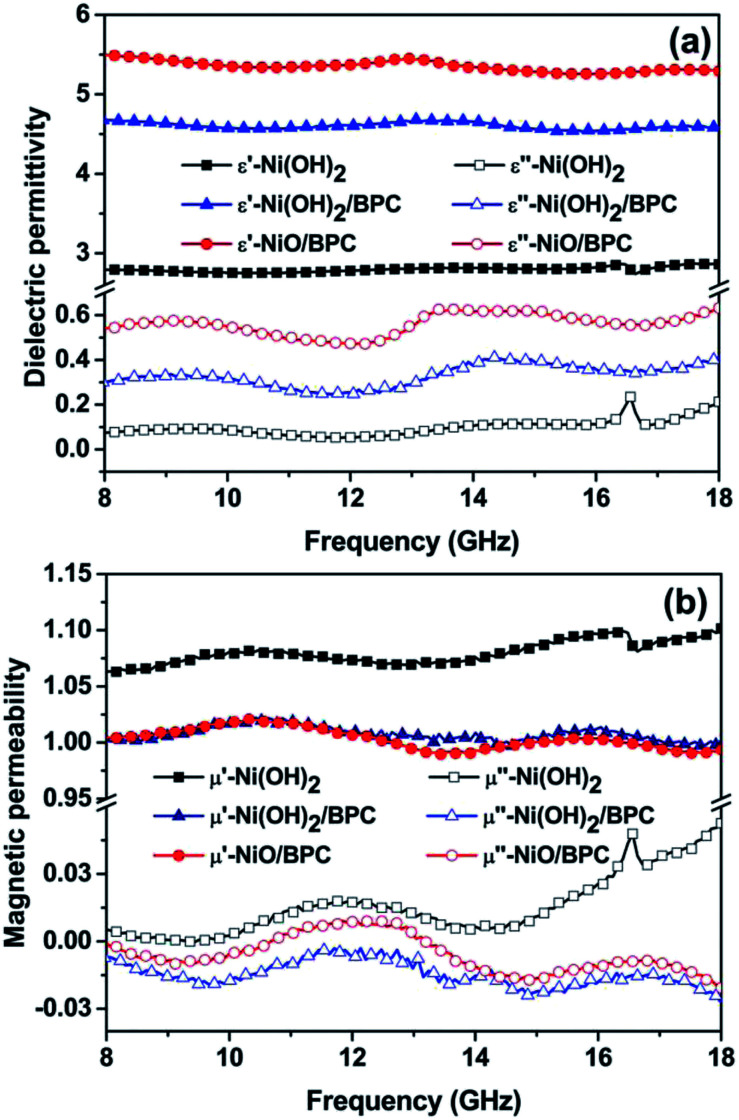
Frequency dependence of complex permittivity (a) and complex permeability (b) of the Ni(OH)_2_, Ni(OH)_2_/BPC and NiO/BPC composites.

For electromagnetic or microwave absorbing materials, there are mainly two critical electromagnetic parameters, *i.e.*, the complex relative permittivity (*ε*_r_) and permeability (*μ*_r_), which highly determine the materials' absorption performances. The real part of permittivity (*ε*′) and permeability (*μ*′) represent the storage capability of electric and magnetic energy, while the imaginary parts (*ε*′′ and *μ*′′) stand for the dissipation capability. To examine the electromagnetic absorption properties of the composites, the *ε*_r_ and *μ*_r_ of the three samples are tested over 8–18 GHz, as shown in Fig. 7. The *ε*′ and *ε*′′ values are almost keeping constant around 2.8 and 0.07 for pure Ni(OH)_2_. Conversely, Ni(OH)_2_/BPC and NiO/BPC composites show an obviously constant real (*ε*′) parts and increasing imaginary (*ε*′′) parts of the permittivity, indicating both high storage capability and dielectric loss. More specifically, the *ε*′ of Ni(OH)_2_/BPC decreases from 4.93 to 4.58, whereas the *ε*′′ value increases from 0.30 to 0.41, with a certain fluctuations over the whole frequency range. In comparison, the *ε*′ of NiO/BPC decreases from 5.95 to 5.29 and the *ε*′′ values increase from 0.58 to 0.63. Therefore, the NiO/BPC composite possess the largest *ε*′ and *ε*′′ values among the three samples, indicating the best dielectric loss. It is widely accepted that *ε*_r_ mainly originates from electronic polarization, interface polarization, ion polarization, on which the structure and morphology exert great important influences.^55,56^ Therefore, it is reasonable to suggest that the larger *ε*′ and *ε*′′ values of Ni(OH)_2_/BPC and NiO/BPC can be related to porous structure with high specific surface areas, leading to more migrating charges and interfacial polarization.^57^

From Fig. 7(b), a similar tendency of *μ*′ and *μ*′′ has been observed for the three samples, showing a regular fluctuation within a narrow frequency range. Particularly, the *μ*′′ value of pure Ni(OH)_2_ shows a slight improvement to 0.05 at 18 GHz. Such phenomenon can be attributed to the uncompensated moments in the nanostructured Ni(OH)_2_ particles, as well as the defects including strains and stacking faults in the interfaces.^58^ After combining with carbon materials, the *μ*′ and *μ*′′ values of both Ni(OH)_2_/BPC and NiO/BPC composites reduced on account of the nonmagnetic characteristics of the carbon matrix. It is necessary to mention that these three samples show nearly the same variation trends and lower values of *μ*′ and *μ*′′ over the entire frequency, indicating the weak magnetic loss properties.

The frequency dependence of dissipation factors including the dielectric loss tangent (tan *δ*_*ε*_ = *ε*′′/*ε*′) and magnetic loss tangent (tan *δ*_*μ*_ = *μ*′′/*μ*′) are calculated based on the measured permittivity and permeability. Clearly, the Ni(OH)_2_/BPC and NiO/BPC composite exhibit a quite higher dielectric loss tangent but a weaker magnetic loss tangent over pure Ni(OH)_2_ in the whole frequency range (Fig. S3†). Especially, the dielectric loss tangent of NiO/BPC composite fluctuates around 0.1 (Fig. S3(b)†) due to the relatively large *ε*′ and *ε*′′ values. Importantly, it is found that the magnetic loss tangents are much smaller than that of the dielectric loss (Fig. S3(b)†), suggesting that the dielectric loss is dominated for the microwave attenuation performances.

Based on the morphological structures, the high surface areas and the electromagnetic loss properties, the microwave absorption performances should be significantly enhanced by coupling Ni(OH)_2_ or NiO with certain carbon materials. Hence, the reflection loss (RL) was calculated to evaluate the microwave absorption capabilities from the measured complex permittivity and permeability through the following transmission line theory.^55,59^1
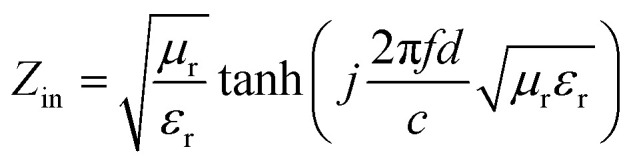
2
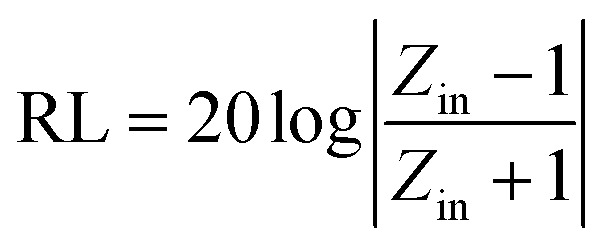
where *Z*_in_ is the normalized input impedance, *c* is the light velocity in free space, *f* is the frequency of the electromagnetic wave and *d* is the thickness of the absorber, respectively. In addition, *ε*_r_ and *μ*_r_ are the relative complex permittivity and permeability, respectively. Lower RL means stronger microwave absorption ability and a RL value of −10 dB represents a 90% absorption of the microwave energy.^60^

**Fig. 8:**
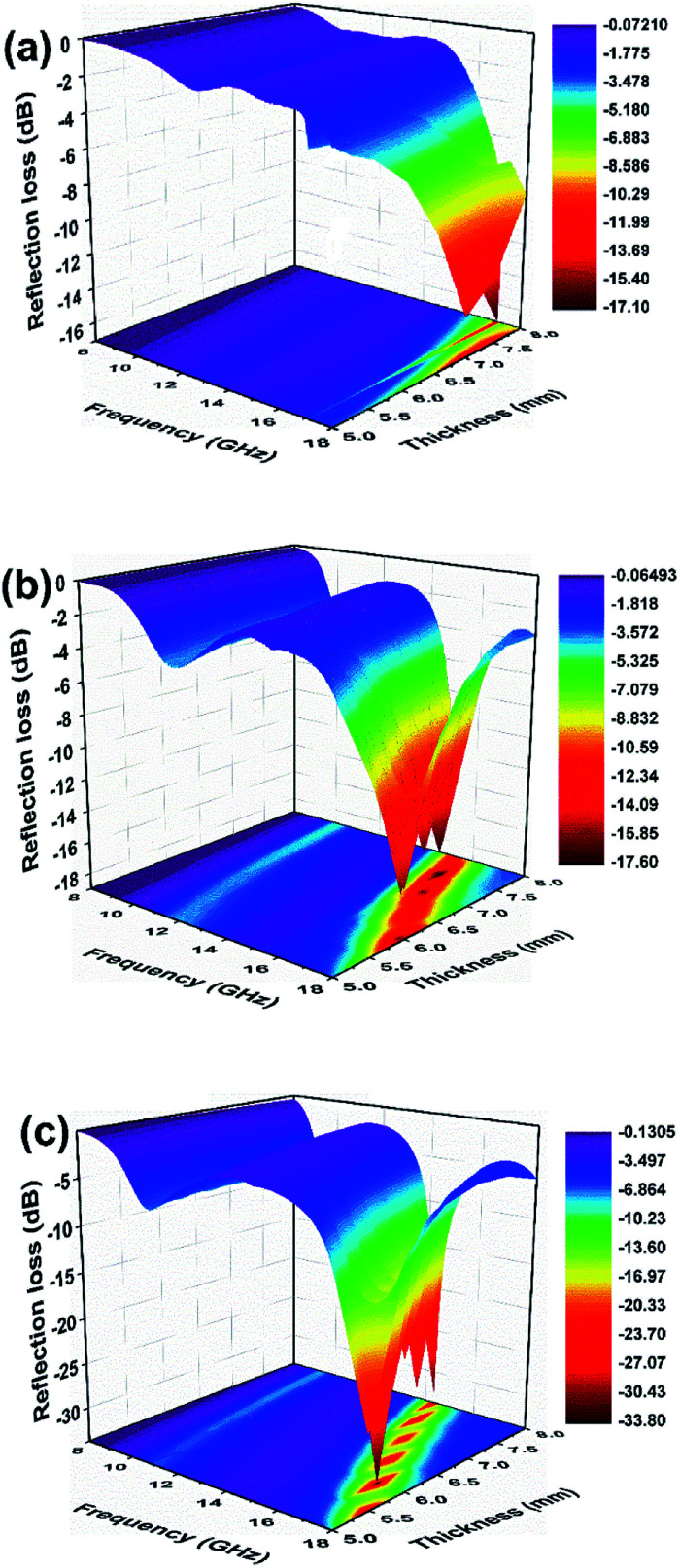
Reflection loss of Ni(OH)_2_ (a), Ni(OH)_2_/BPC (b) and NiO/BPC (c) composites at various thicknesses.

The RL values of the as-prepared samples are simulated at different thicknesses, as summarized in Fig. 8. From Fig. 8(a), pure Ni(OH)_2_ shows weak microwave absorption ability in terms of poor reflection loss and narrow EAB. In contrast, Ni(OH)_2_/BPC and NiO/BPC composites exhibit distinctly microwave absorption properties with a broader absorption bandwidth. This enhancement could be reasonably attributed to the improvement of the complex relative permittivity and dielectric loss derived from interfacial polarization and the dipole polarizations owing to the coupling effect of carbon materials.^61,62^ From Fig. S4(a and c),† it clearly shows that pure BPC has superior complex relative permittivity and dielectric loss. However, Fig. S4(d)† shows that pure BPC has a poor microwave absorption ability and the maximum RL value is only −2.98 dB at an optimal sample thickness of 1 mm. When the thickness is further increased, its RL decreases due to the large dielectric loss abilities. However, Ni(OH)_2_/BPC composite exhibits a maximum reflection loss of −17.6 dB with a broaden EAB of 1.5 GHz, as displayed in Fig. 8(b). NiO/BPC composite shows a highly desirable microwave absorption performance with maximum reflection loss of −33.8 dB. Moreover, an effective absorption bandwidth of 2 GHz is obtained when the thickness varies from 6 mm to 8 mm (Fig. 8(c)).

**Fig. 9:**
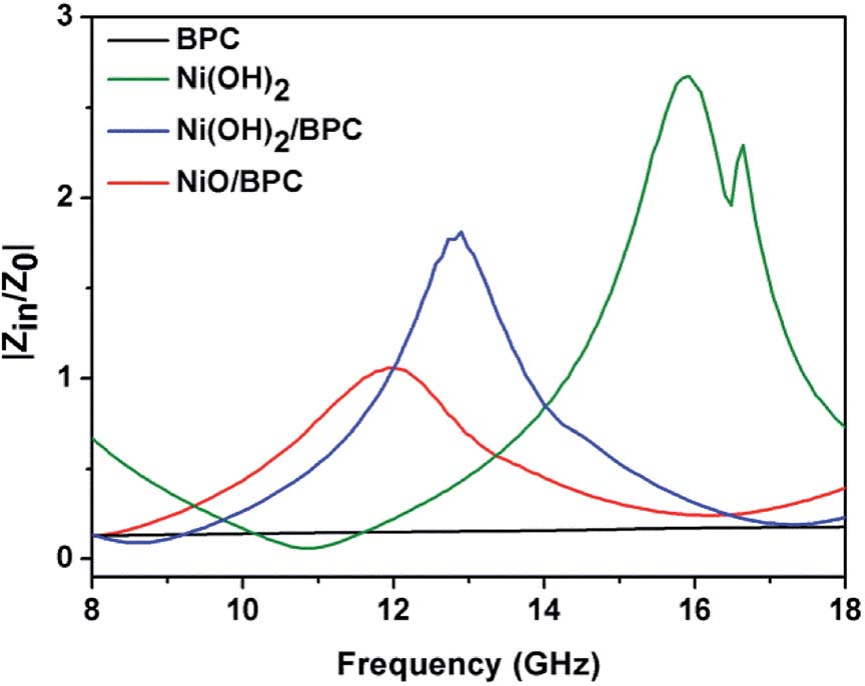
Modulus of normalized input impedance *Z* of BPC, Ni(OH)_2_, Ni(OH)_2_/BPC and NiO/BPC composites with 8 mm.

It is widely accepted that the impedance matching behaviour is prerequisite to achieve outstanding microwave absorption properties. To achieve an ideal match, the *ε*_r_ and *μ*_r_ of the EAM should be as close as possible, in which most of incident microwave could propagate into absorber and realize subsequent dissipation.^63^ The value of *Z* = |*Z*_in_/*Z*_0_| was obtained by means of eqn (1) with thickness of 8 mm, where the completely impedance matching will be gained when *Z* = 1. It is found in Fig. 9 that NiO/BPC composite possess ideal matching conditions, where the matching frequency is 12.2 GHz and the minimum RL (−33.8 dB) can be achieved. However, the *Z* values at same thickness for other samples are far away from 1. It is worth to note that the *Z* values of pure BPC are almost kept constant as 0.1. This phenomenon could arise from the higher complex relative permittivity and lower permeability of BPC (Fig. S4†). Hence, the excellent microwave absorption properties of NiO/BPC could be attributed to the favourable impedance matching.

**Fig. 10:**
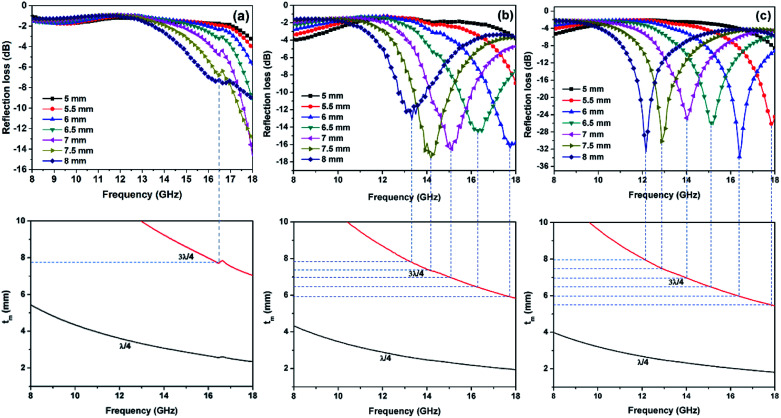
Dependence of matching thickness (*t*_m_) on matching frequency (*f*_m_) of three samples based on the *λ*/4, and 3*λ*/4 models.

It should be point out that all the absorbing peaks are shifted towards lower frequency with the increase of the absorber thickness. This phenomenon can be explained by the quarter-wavelength equation model.^64^3
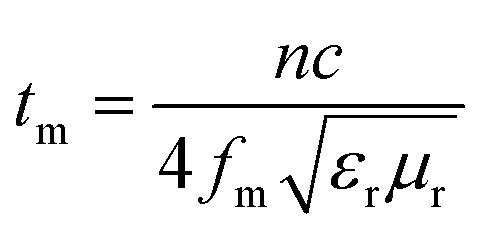
where *f*_m_, *t*_m_ and *c* are the matching frequency, the matching thickness and velocity of light, respectively. Obviously, the optimal peak frequency is inversely proportional to the thickness of absorbers. As detected in Fig. 10, the matching thickness for the effective absorption peak is well consistent with the actual calculation layer thicknesses. Besides, the large differences in microwave absorption properties between Ni(OH)_2_/BPC and NiO/BPC can be explained by the complex relative permittivity differences.

As is already known, microwave absorption performances of absorbing materials are closely associated with their dielectric loss, magnetic loss and impedance matching behaviours. First of all, impedance matching must be fulfilled to achieve high microwave absorption efficiency. It is easy to understand that the incident electromagnetic wave should effectively propagate into the absorbers, and so the attenuation can be considered further. Secondly, the electromagnetic wave should be dissipated as much as possible; otherwise the absorber will be electromagnetically transparent. As to the NiO/BPC composite, on the one hand, the small values of relative permittivity and permeability can endow it a better impedance matching between the space and the interface of the sample (as shown in Fig. 9), guiding the incident wave to penetrate into the interior of the material. On the other hand, the hierarchically porous structural property of the NiO/BPC can be able to increase additional transmission tunnels of the transmitting waves. So it can induce multiple reflection and scattering of the electromagnetic wave to enhance the attenuation capacity, and thus exhibit better electromagnetic wave absorption properties.^65,66^ In other words, both the porous structure and the long-range cross-linked transmission network are imperative to the excellent microwave absorption performance of the NiO/BPC composite, as that has been proved in the graphene foam systems.^67^

To further interpret the dielectric behaviours based on electromagnetic theory, Cole–Cole semicircles of the as-prepared samples were analysed. According to Debye dipolar relaxation, the relative complex permittivity can be explained by the following equation,^68^4
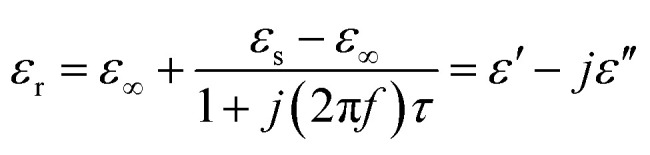
where *ε*_s_ and *ε*_∞_ are the static permittivity and relative dielectric permittivity at infinite frequency, respectively, and *τ* is the polarization relaxation time.

From eqn (4), *ε*′ and *ε*′′ can be calculated in the following,5
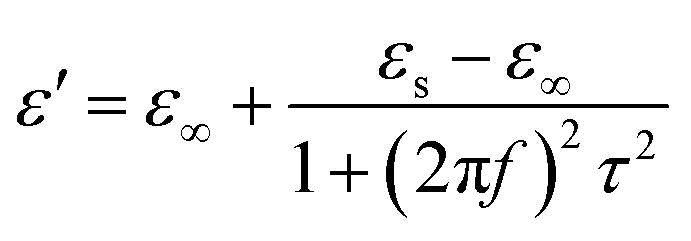
6
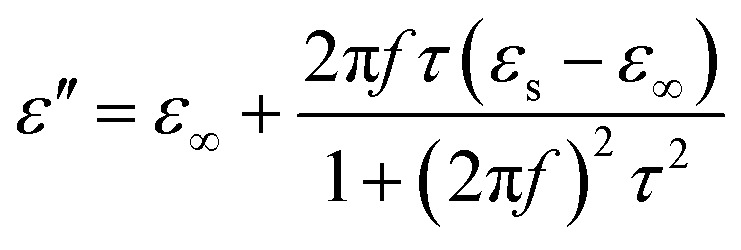


So, the relationship between *ε*′ and *ε*′′ can be obtained,

7 (*ε*′ − *ε*_∞_)^2^ + (*ε*′′)^2^ = (*ε*_s_ − *ε*_∞_)^2^

Thus, the plot of *ε*′ *versus ε*′′ would form a single semicircle, which is generally denoted as the Cole–Cole semicircle, in which each semicircle is assigned to one Debye relaxation process. As shown in Fig. S5(a),† pure Ni(OH)_2_ shows a completely disordered curve, indicating no obvious dielectric relaxation process. However, several distinguishable Cole–Cole semicircles are observed with respect to Ni(OH)_2_/BPC and NiO/BPC composites, implying the complicated dielectric relaxation processes. Furthermore, the distorted Cole–Cole semicircle also indicates the probable existence of other dissipation except for the dielectric relaxation, such as conductance loss, space charge polarization and interfacial polarization.^67^ The big curves in Fig. S5(b) and (c)† can be attributed to the conduction loss caused by the electric conductivity of the composites. In addition, the composites possess a large number of interfaces, and there are enormous defects, which can cause an accumulation of space charges and thus results in the formation of space charge polarization and other additional relaxation processes.^69,70^ In a whole, the larger specific surface areas of Ni(OH)_2_/BPC and NiO/BPC composites will provide massive active sites to induce multiple reflection and scattering of electromagnetic waves, along with the dipole polarizations owing to external defects on the surface. Besides, NiO/BPC composites possess more Cole–Cole semicircle, which may be attributed to the clustered defects on NiO surfaces caused by the existence of oxygen vacancies. The defects could break the balance of the charge distribution and contribute to the dipole polarization and related relaxations.^71^ Under altering electromagnetic field, those can increase energy dissipation and further enhancing microwave absorption performance of NiO/BPC composites. As a whole, the as-synthesized NiO/BPC can be considered as a kind of microwave absorbing candidates.

## 4. Conclusions

In summary, porous composite materials of Ni(OH)_2_/BPC and NiO/BPC derived from pine nut shells were fabricated by a feasible KOH activation and subsequent water-bathing precipitation method. After combined with carbon material, the NiO/BPC composite exhibits excellent microwave absorption performances with a maximum reflection loss value as −33.8 dB at 16.4 GHz and the effective absorption bandwidth of about 6.7 GHz (from 11.3 to 18.0 GHz). The introduction of biomass porous carbon leads to higher permittivity and improved dielectric loss, which can be ascribed to Debye dipolar relaxation, electron polarization relaxation and interfacial polarization. In addition, the porous structure with larger surface areas may induce multiple reflections and scattering of the incident microwaves and energy dissipation. Therefore, it is considered that the NiO composite incorporated with biomass porous carbon provides a new strategy for electromagnetic absorbing materials due to great potential in high-capability, lightweight and the renewability, easy mass production of carbon sources.

## Conflicts of interest

The authors declare no competing financial interest.

## Supplementary Material

RA-009-C9RA00466A-s001
