# Supplementary material for: Biomass carbon derived from pine nut shells decorated with NiO nanoflakes for enhanced microwave absorption properties
Source: RSC Adv. 2019 Mar 19;9(16):9126–35. doi: 10.1039/c9ra00466a (PMC9061977; doi:10.1039/c9ra00466a)
Supplement: RA-009-C9RA00466A-s001 [file RA-009-C9RA00466A-s001.pdf]

Electronic Supplementary Material (ESI) for RSC Advances.
This journal is © The Royal Society of Chemistry 2019

Supplementary materials

Biomass carbon derived from pine nut shells decorated with NiO nanoflakes for enhanced microwave absorption properties

Huiya Wang[a], Yanlin Zhang[a], Qiuyue Wang[a], Chaowei Jia[a], Pan Cai[a], Gang Chen[a,b], Chengjun Dong*[a,b], Hongtao Guan*[a,b]

[a.] *School of Materials Science and Engineering, Yunnan University, Kunming 650091, P. R. China.*

[b.] *Yunnan Province Key Lab of Micro-Nano Materials and Technology, Yunnan University, Kunming 650091, P. R. China.*

* Corresponding author: dongchjun@hotmail.com (CJ Dong), htguan06@ynu.edu.cn (HT Guan)

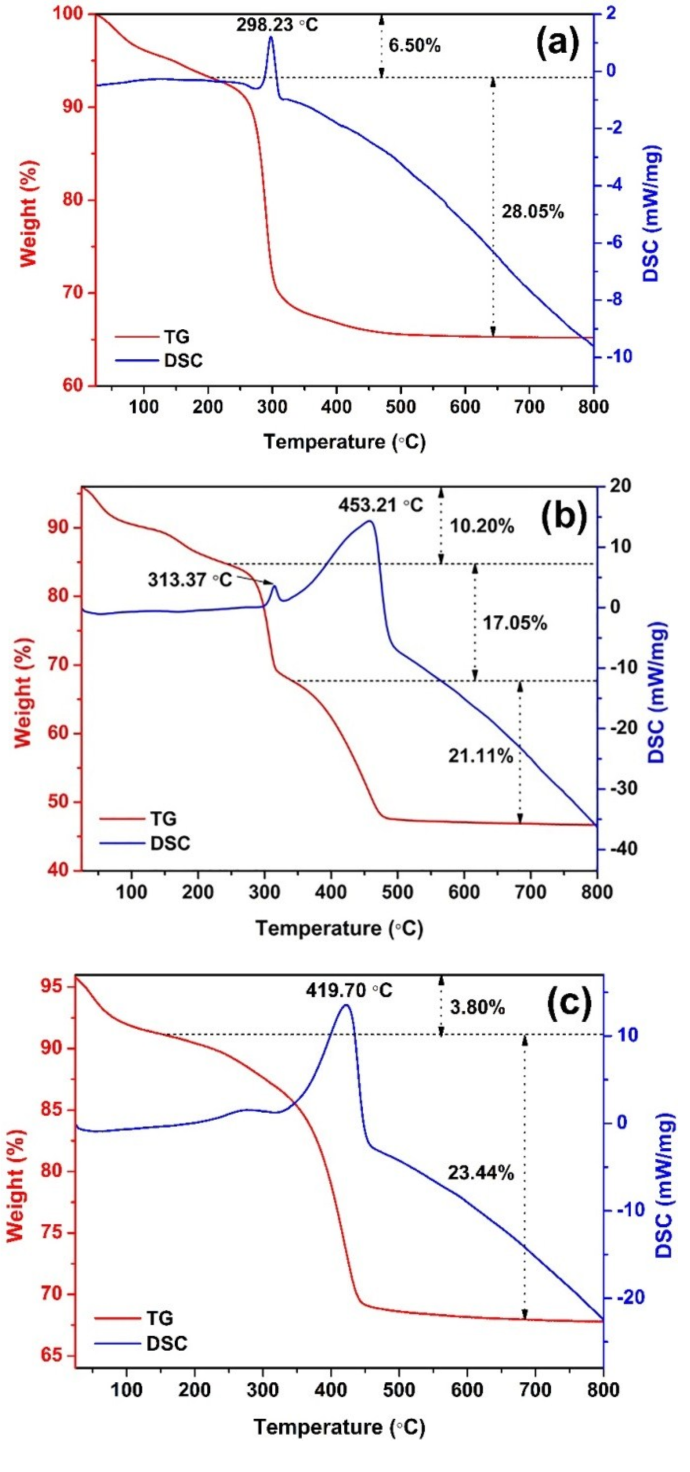

Fig. S1. TG-DSC curves of pure $Ni(OH)_2$ (a), $Ni(OH)_2$/BPC (b) and NiO/BPC (c) composites at a heating rate of 5 ºC/min from room temperature to 800 ºC in air.

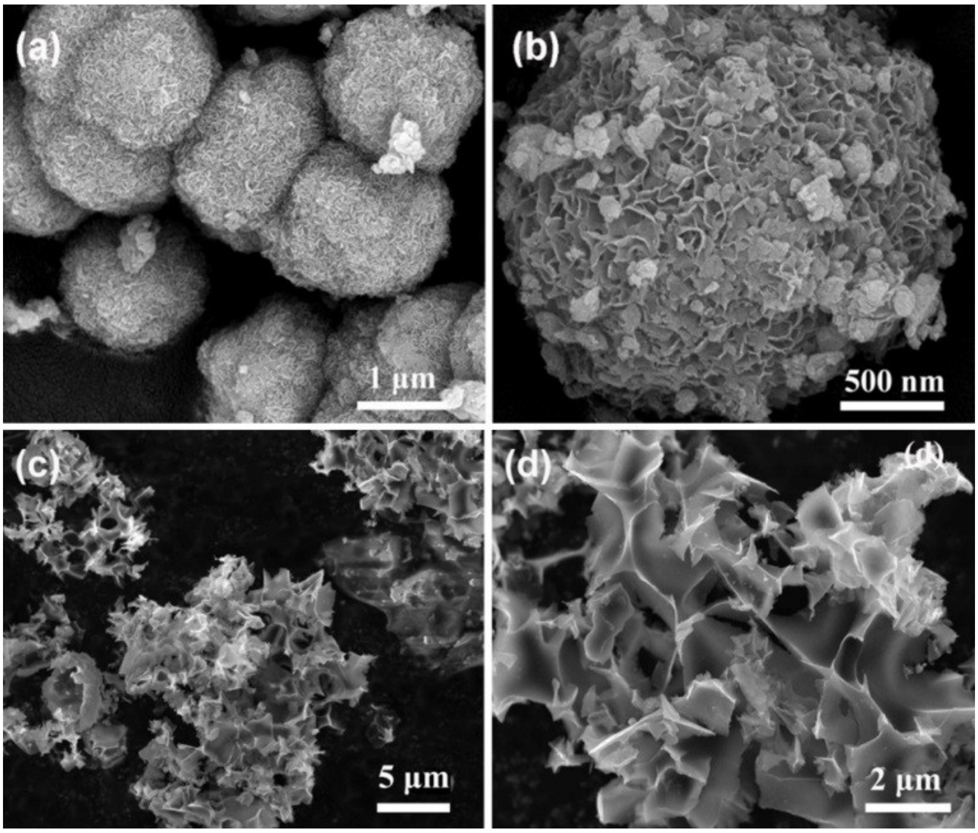

Fig. S2. SEM images of pure $Ni(OH)_2$ nanostructures (a, b) and biomass activated carbon (c, d).

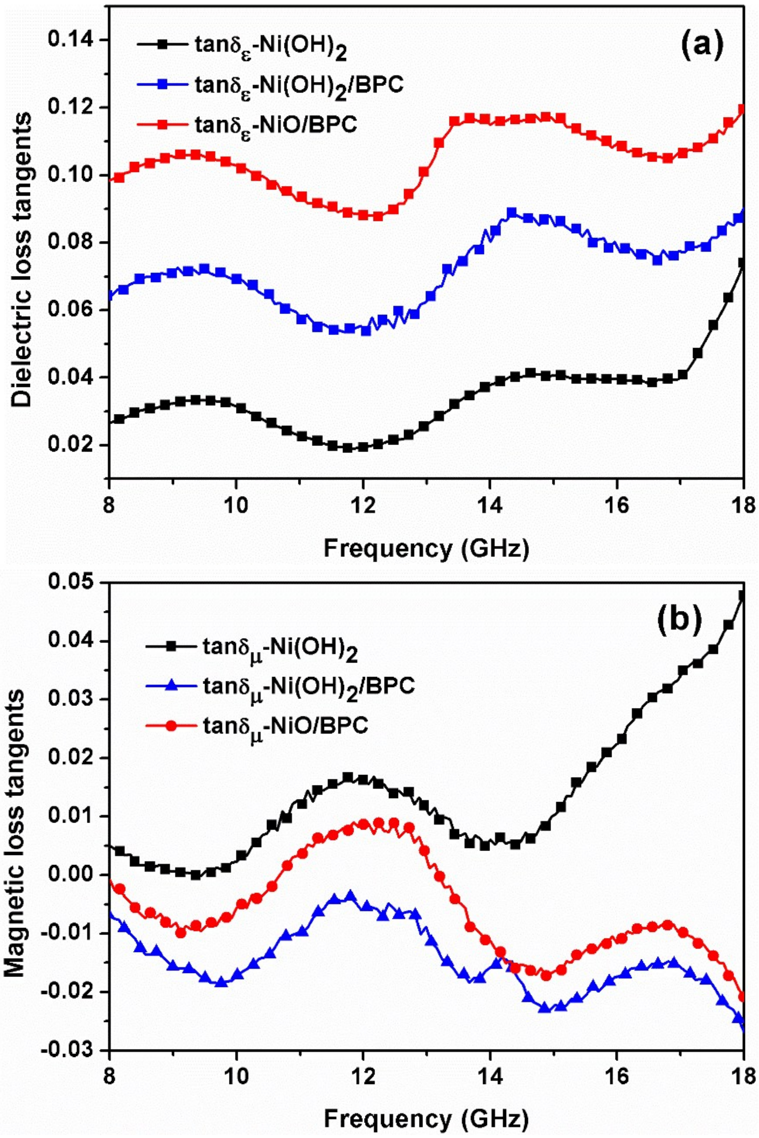

Fig. S3. Dielectric (a) and magnetic (b) dissipation factors of the $Ni(OH)_2$, $Ni(OH)_2$/BPC and NiO/BPC compositions.

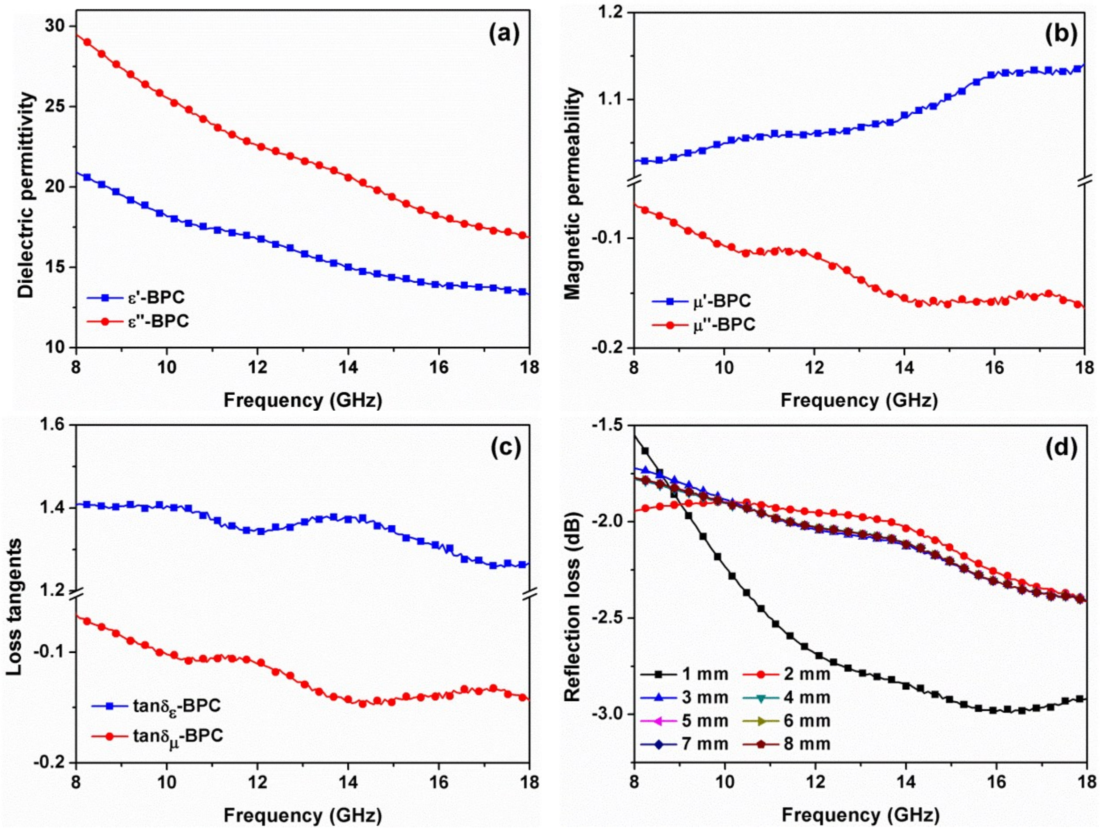

Fig. S4. Dielectric permittivity (a), magnetic permeability (b), dissipation factors (c) and reflection loss (d) of the BPC sample.

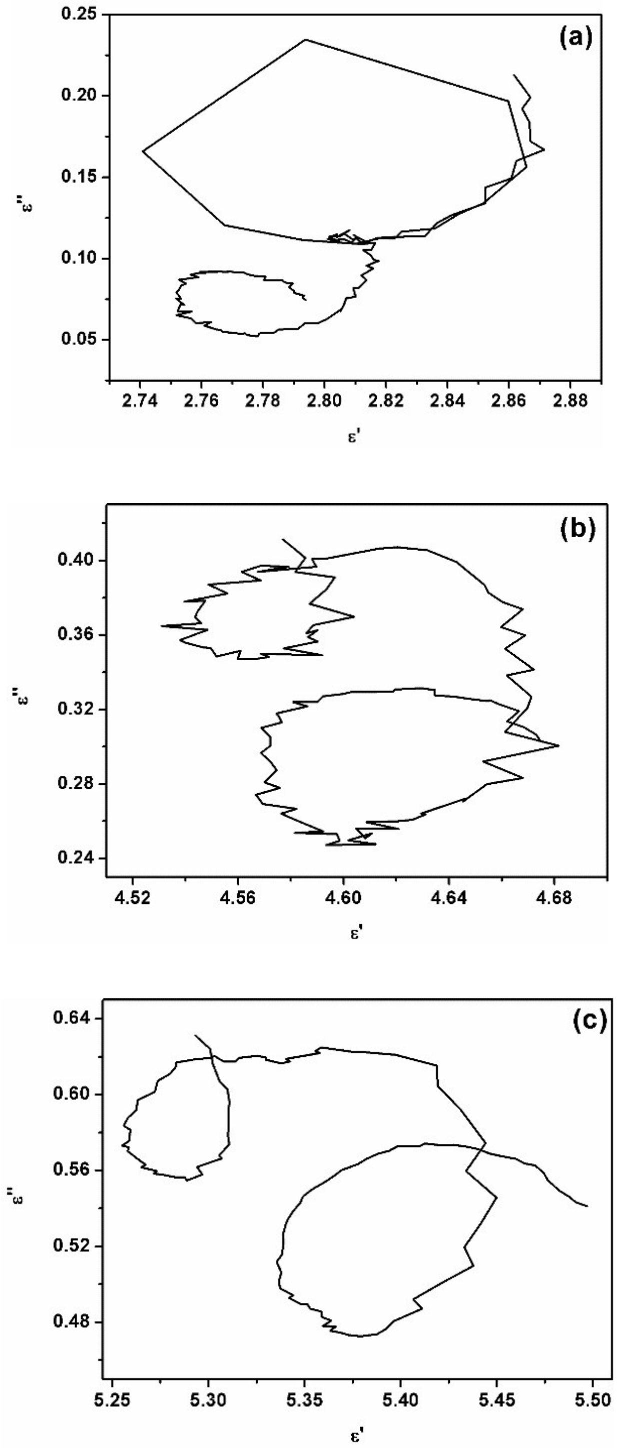

Fig. S5. Cole-cole plots of the $Ni(OH)_2$ (a), $Ni(OH)_2$/BPC (b) and NiO/BPC (c) composites.
